# Recent advances in the understanding and treatment of acute myeloid leukemia

**DOI:** 10.12688/f1000research.14116.1

**Published:** 2018-08-06

**Authors:** Justin Watts, Stephen Nimer

**Affiliations:** 1 Sylvester Comprehensive Cancer Center and Miller School of Medicine, University of Miami, Miami, Florida, USA

**Keywords:** acute myeloid leukemia (AML); genomics; epigenetics; targeted therapies; drug development

## Abstract

Acute myeloid leukemia (AML) is a clinically and genetically heterogeneous disease that has a poor prognosis. Recent advances in genomics and molecular biology have led to a greatly improved understanding of the disease. Until 2017, there had been no new drugs approved for AML in decades. Here, we review novel drug targets in AML with a focus on epigenetic-targeted therapies in pre-clinical and clinical development as well as the recent new drug approvals.

## Introduction

While the past 10 to 20 years witnessed an explosion in the number of US Food and Drug Administration (FDA)-approved therapies for lymphoid malignancies, myeloid drug development lagged behind, and few drugs were approved, particularly for acute myeloid leukemia (AML). This was puzzling in light of similar, if not greater, advances in the understanding of the genetic basis and pathophysiology of myeloid malignancies, which represent about 5% of all adult cancers ^1^. The challenge of translating these scientific discoveries into effective therapies for patients with AML or myelodysplasia constituted an urgent unmet medical need. However, the FDA has recently approved three new drugs for AML and re-approved gemtuzumab ozogamicin (GO), all in 2017, offering encouragement that more breakthroughs are coming, commensurate to our biological understanding of myeloid neoplasia. This review focuses on AML pathogenesis and recent therapeutic advances, including the genetic heterogeneity of AML, the critical role that epigenetic abnormalities play in its development, promising pre-clinical drug targets and ongoing clinical trials, and the recently approved new agents.

## Background

AML is primarily a disease of older adults with a median age at diagnosis of about 70 years ^1, 2^. It is the most common leukemia after chronic lymphocytic leukemia and is the leading cause of leukemia-related deaths in the US ^1^. Overall, AML is fatal for the majority of patients (~80%), and modern cooperative group studies show that patients younger than 55 to 60 years of age have about 40% 5-year survival but that those older than 60 have an abysmal less than 5% to 10% 5-year survival ^1– 4^. Until very recently, the standard chemotherapy for AML remained largely unchanged for decades, and the survival improvements over time in younger patients were attributable mostly to better supportive care and safer/improved hematopoietic stem cell transplantation (HSCT) techniques ^4, 5^. Outcomes have remained particularly poor and without significant improvement in older adults since the 1970s ^6^. In addition to host factors, adverse biological features are common in older patients, making AML a challenge to treat in this patient population. Consequently, we have seen rising AML mortality rates as the aging population continues to increase ^5– 7^ despite some improvements in the treatment of younger patients. Clearly, new and better therapies are urgently needed for AML, particularly for older patients.

The clinical responses seen with all- *trans* retinoic acid (ATRA) in acute promyelocytic leukemia (APL), subsequently found to reflect the presence of the PML-RARα translocation fusion product, and the incredible success of imatinib in t(9;22) chronic myeloid leukemia (CML), driven by the BCR-ABL tyrosine kinase, led many to believe that identifying single genetic abnormalities in myeloid cancers would predict response to targeted therapies. However, we now know that, in most clinical settings, only a small fraction of patients will respond to targeted therapies. For example, we know very well that only some AML patients with *FLT3*-internal tandem duplication ( *FLT3*-ITD) mutations respond to kinase inhibitors, only some with del(5q) myelodysplastic syndromes (MDSs) respond to lenalidomide, and only some with isocitrate dehydrogenase 2 ( *IDH2*) mutations respond to IDH2 inhibitors. In many cases, even patients who initially respond to targeted therapies rapidly develop resistance. This is likely due to a variety of factors, including epigenetic changes and the type and number of co-occurring genetic mutations. In this review, we will present these challenges and how to potentially solve them.

## Genetic heterogeneity and epigenetic deregulation in acute myeloid leukemia

While the total number of genetic mutations and frequency of chromosomal aneuploidy are less in AML than in most solid tumors, AML remains a remarkably genetically heterogeneous disease ^8^. Diverse combinations of specific driver mutations have a clear effect on prognosis. In a landmark study, Patel *et al*. reported that a comprehensive assessment of the major co-occurring mutations in AML significantly impacts prognosis and treatment ^9^. Indeed, this work, plus decades of laboratory and clinical research, has convincingly demonstrated that it is the constellation of mutations in AML that determines prognosis and responsiveness to therapy. Considering this fundamental principle, translational researchers have created mouse models combining these different lesions, such as those expressing AML1-ETO (AE) in *Tet2* ^+/–^ cells or *Asxl2* ^+/–^ cells or expressing FLT3-ITD in *Tet2* ^+/–^ cells in order to better reflect and determine which treatments may work in which AML patient subtypes.

Another seminal paper recently described 11 unique genomic/mutational classes of AML, with additional (non-class-defining) driver mutations co-occurring within each class, such as *FLT3*-ITD, resulting in dozens of AML subtypes which may each require a unique multi-agent therapy ^10^. Furthermore, in addition to DNA sequence mutations, AML has been increasingly recognized as an epigenetically driven disease. While most patients have at least one mutation in an epigenetic modifying protein, essentially all patients with AML have deregulated epigenetic regulation of multiple pathways as a hallmark ^11, 12^. Because the effect of combinatorial genetic mutations on chromatin structure and DNA methylation is not simply additive, accurate, disease-predicting, pre-clinical models remain extremely valuable as we attempt to increase the therapeutic index of novel, epigenetic-focused treatments. Combinations of mutations clearly interact to drive the initiation and progression of AML and may create unique sensitivities to epigenetic-focused and other targeted or chemo-therapies. For example, co-occurring *IDH2* and *DNMT3A* mutations result in a distinct DNA methylation pattern different from what is seen with either mutation alone (“epigenetic antagonism”), leading to upregulation of Ras signaling and unique sensitivity to MEK inhibition in *IDH2*/ *DNMT3A* double-mutants ^11^. Multifaceted studies that can define epigenetic or gene expression signatures that are associated with responsiveness to specific therapies should translate into improved therapies for patients with myeloid malignancies.

Though clinically heterogeneous, a wealth of genome-wide studies point to epigenetic deregulation as the major driver of the myeloid malignancies ^8– 10^. In fact, we now know that mutations in *ASXL1*, *ASXL2*, *TET2*, *DNMT3A*, and *IDH1/2* occur more commonly in MDS and AML than do chromosomal translocations, which also target key transcription factors and epigenetic regulators. The reversibility of epigenetic changes, unlike that of genetic changes, provides an opportunity to potentially treat virtually all leukemias (and cancers) using epigenetic-focused approaches. Given the speed at which epigenetic-focused therapeutics are being formulated, accurate pre-clinical models that can identify predictive molecular signatures and carefully constructed clinical trials of epigenetic-focused agents in rigorously defined patient populations are greatly needed. To achieve success, we need to continue to create new reagents and more accurately model the multitude of gene mutations found in patients with AML, MDS, and MDS/myeloproliferative neoplasm (MPN).

This review will focus on the promise of epigenetic therapies but also on novel chemotherapy agents, antibody-drug conjugates, and kinase inhibitors that either are FDA approved or appear promising in clinical trials. However, it is the combination of epigenetic agents used together or with other agents that holds the most promise of making AML a more successfully treatable disease, like APL and CML.

Additionally, while inhibition of overexpressed/disease-promoting—for example, enhancer of zeste homolog 2 (EZH2), protein arginine methyltransferases (PRMTs), and lysine-specific demethylase 1 (LSD1)—and neomorphic/gain-of-function epigenetic enzymes (such as IDH1/2) is clearly important, the predominantly decreased activity of *mutated* epigenetic enzymes in myeloid diseases provides at least three additional unique therapeutic possibilities that have been understudied to date: (1) successfully restoring normal enzymatic function by boosting the function of the remaining functional allele; (2) triggering the complete loss of enzymatic function, triggering cell death, which may be more readily accomplished in cells with only one normal allele; and (3) deleting an epigenetic gene to create a unique sensitivity to manipulating targets in an interacting pathway. Exploring these possibilities represents a rapidly evolving new area of AML research.

## Targeting epigenetics in acute myeloid leukemia (pre-clinical drug targets)

AML is a clinically heterogeneous disease driven by diverse pathogenic lesions, including somatic mutations, chromosomal defects, and epigenetic changes observed in primary patient samples. Epigenetic deregulation, which is due in part to somatic mutations in epigenetic regulators, is a hallmark of the pathogenesis of myeloid malignancies. The reversibility of epigenetic changes, even when driven by underlying genetic alterations, suggests that improvements in outcome should be achievable by tailoring treatments to the underlying epigenetic defects. In this section, we will review novel epigenetic targets being studied in the laboratory in order to rapidly identify new therapies for patients. We already have clinical evidence that epigenetic therapies are active in AML. However, given the complexity of epigenetic interactions in AML and the number of potentially co-occurring sensitizing or resistance-conferring mutations, often within the same patient or even the same cell, and potentially unanticipated drug effects on epigenetic regulation, combination AML therapy with different classes of drugs will likely remain the hallmark of AML treatment but with more epigenetic-targeted agents being incorporated into existing and emerging treatment paradigms.

### TET2

TET2 is a dioxygenase enzyme using alpha-ketoglutarate (αKG), Fe ^2+^, and O _2_ for hydroxylation of DNA methylcytosine (5mC), converting it into 5-hydroxymethylcytosine (5hmC), which can be converted into other derivatives, including ultimately demethylated DNA. *TET2* is one of the most commonly mutated genes in myeloid malignancies, and *TET2* mutations are also found in apparently hematologically normal individuals with clonal hematopoiesis ^13, 14^. *TET2* mutations are commonly an ancestral mutational event and, as such, constitute a suitable target for intervention in the early stages of hematopoietic stem cell/hematopoietic progenitor cell (HSC/HPC) expansion. *Tet2* loss/haploinsufficiency in HSCs/HPCs leads to MDS/MPN development in mice and cooperates with other disease alleles (for example, AML1-ETO or *FLT3*-ITD) to induce AML ^15– 18^. Specifically, *Tet2* loss leads to increased HSC self-renewal and skewed differentiation toward the monocytic lineage ^14, 15^. TET2 catalytic activity is required for its function as a myeloid leukemia tumor suppressor in HSCs/HPCs ^17^.

*TET2* mutations are mostly heterozygous, resulting in enzymatic haploinsufficiency, leading to decreased 5hmC and concomitantly increased 5mC. Therefore, a compelling therapeutic strategy is to augment TET2 function by increasing the enzymatic activity of the remaining wild-type allele. To split O _2_ for dioxygenation, TET2 requires three electrons: two from αKG and one from vitamin C ^15^. Agents such as dimethyl 2-oxoglutarate (DMKG) and vitamin C augment TET2 enzymatic activity *in vitro* and in mouse models and hold the potential to restore TET2 activity ^19^. The phenotype of Tet2 restoration, which is mimicked by vitamin C treatment, includes reversal of aberrant hematopoietic stem and progenitor cell (HSPC) self-renewal and restoration of hypomethylation, differentiation, and cell death ^19^. Activators of TET2 catalytic activity, such as vitamin C, are capable of reversing the leukemic-transforming ability of *TET2* haploinsufficiency and thus represent promising combination partners with other therapies, such as DNA hypomethylating agents (HMAs) and p300 inhibitors ^19^. Cimmino *et al*. have also shown that TET-mediated DNA oxidation induced by vitamin C treatment in leukemia cells may enhance their sensitivity to poly (ADP-ribose) polymerase (PARP) inhibitors ^19^. Combining vitamin C with HMAs in *TET2*-mutant (TET2m) MDS to augment their therapeutic potential is now being studied in the clinic. Clinical trials using high-dose intravenous vitamin C in TET2m MDS, with or without concomitant HMA therapy, are now available (ClinicalTrials.gov Identifier: NCT03433781) and this represents an exciting new therapeutic strategy given that TET2m patients already have increased sensitivity to HMAs ^20^. Given that *TET2* clonal hematopoiesis of indeterminate potential (CHIP) mutations affect older adults (~5%) ^13^, the importance of developing TET2-targeted therapies will only become more relevant as the population ages and the incidence of both *de novo* and therapy-related MDS continues to rise.

### ASXL1

The Drosophila additional sex-combs (ASX) gene is a chromatin-binding polycomb protein required for embryogenesis. Genomic studies have highlighted that the ASXL (additional sex combs-like) family members ASXL1 and ASXL2 are recurrently mutated in human AML. ASXL1 is mutated (deleted or truncated) at high frequencies in all forms of myeloid malignancy ^21, 22^; it is associated with poor prognosis in patients with chronic myelomonocytic leukemia (CMML), MDS, myelofibrosis, and AML ^23, 24^. Thus, therapies targeting mutant *ASXL1*-bearing cells are sorely needed. In contrast, ASXL2 is specifically mutated in t(8;21) AML ^25^. Importantly, ASXL1 and ASXL2 mutations occur in a mutually exclusive fashion in t(8;21) AML and, owing to their similarities, it has been hypothesized that there is a shared mechanism of leukemogenesis arising from these two mutations. However, it is still not clear whether mutations in *ASXL1*/ *2* confer loss-of-function, dominant-negative activity or generate a novel or altered function. Thus, their shared or unique mechanisms in leukemogenesis remain unclear. The majority of the *ASXL1* mutations are nonsense/frameshift and heterozygous and expected to be loss of function. However, a truncated ASXL1 protein (C-terminus) has been shown to be expressed in cells harboring *homozygous* mutations of *ASXL1*, suggesting dominant-negative or even gain-of-function activity ^26^. Whether mutations in *ASXL1* generate haploinsufficiency or dominant-negative or gain-of-function proteins (or combinations thereof) is a question with great clinical implications given the highly recurrent and adverse nature of *ASXL1* mutations in MDS/AML.

To this end, various groups have generated model systems to dissect the possible roles of ASXL1/2 alterations in leukemogenesis and identify therapeutic vulnerabilities they may create ^27, 28^. Avenues for identifying drug candidates for *ASXL1*/ *2*-mutated myeloid malignancies include studying the biological effects of *ASXL1* versus *ASXL2* loss in the presence of cooperating mutations such as AML1-ETO.

### Lysine acetyltransferases

The CBP/p300 coactivator family of histone lysine acetyltransferases (KATs) is another emerging and promising target in myeloid malignances. KATs—and, in particular, CBP/p300—have been shown to play important tumor suppressor and oncogenic roles in cancer ^29, 30^. Whereas histone deacetylases have been targeted therapeutically in patients with myeloid neoplasms (with modest success), the KAT enzymes have been largely ignored. Since the first nuclear KAT enzyme was isolated 20 years ago ^31^, exciting data have emerged implicating histone (and non-histone) protein acetylation in regulating gene expression and the pathways that control normal and neoplastic cell growth. CBP and p300 are two related, broadly expressed KAT proteins that have been shown to affect normal HSC self-renewal and differentiation ^29^. Mutations in the *EP300* ( *KAT3B*) and *CBP* ( *KAT3A*) genes have been found in lymphoma, small cell lung cancer, and other solid tumors, identifying their tumor suppressor function ^32^. p300 can also function as an oncogene in AML ^33^, melanoma ^34^, and prostate cancer ^35^. In fact, there appear to be distinct roles for p300 in mouse models of myeloid malignancies: an oncogenic role in AML1-ETO-driven AML ^33^ and a tumor suppressor role in MDS driven by the NUP98-HOXD13 (NHD13) fusion protein ^36^. Consequently, p300 may display variable activity, depending on the disease that it initiated. The cell context-specific functions of p300 suggest that developing both agonist and antagonist drugs may be therapeutically relevant.

***CBP/p300 inhibitors.*** In 2011, we identified the critical role of p300 in binding to AML1-ETO and the subsequent acetylation of AML1-ETO on K43, which regulates the ability of AML1-ETO to activate gene expression, promote self-renewal, and trigger AML ^33^. We showed that both pharmacologic inhibition and genetic knockdown of p300 improve the survival of AML1-ETO-bearing mice and that CBP/p300 inhibitors impair the growth of AML1-ETO-expressing (and several other) human leukemia cell lines ^33^. This suggests that p300/CBP may be critical for leukemia cell self-renewal and that targeting its catalytic activity may have broad therapeutic potential in AML1-ETO-positive AML. The first selective CBP/p300 inhibitor (Lys-CoA) was developed in the 1990s ^31^, and newer-generation, more potent CBP/p300 inhibitors are in active development and are being studied in pre-clinical cancer models ^37^. Salicylate derivatives and related anti-inflammatory drugs such as diflunisal have also been demonstrated to inhibit both CBP and p300 and shown anti-leukemic activity in pre-clinical AML models ^38^.

***CBP/p300 activators.*** Despite the rarity of the t(2;11)(q31;p15) in MDS, NUP98-HOXD13-driven MDS mice have been studied since 2005 because they accurately reflect the clinical behavior of human MDS ^39, 40^. This model has been used by us and others to study the role of p53 ^40^, Msi2 ^41^, and p300 ^33, 36^ in MDS. Notably, deletion of p300 (but not CBP) markedly accelerated leukemogenesis in the setting of NHD13-driven MDS, as 100% of the NHD13 ^+^, p300 null mice died from AML within 20 weeks ^36^. Although NHD13 ^+^ bone marrow (BM) cells demonstrated defective *in vitro* growth, which is characteristic of MDS, the absence of p300 promoted the growth and self-renewal of these cells despite having little or no effect on normal mouse BM cells. The underlying mechanisms are being actively pursued using this and other mouse models of MDS/AML. Additionally, p300 activators are being investigated in these models. One candidate compound is I-CBP112, a selective and potent p300/CBP bromodomain inhibitor ^42^ that unexpectedly *stimulates* the acetylation of nucleosomes by both p300 and CBP. Therefore, exposure to I-CBP112 increases H3K18Ac in multiple cancer cell lines ^43^. I-CBP112 is being assessed for anti-growth properties in myeloid malignancies with subnormal p300/CBP function. Ongoing studies will assess whether restoring p300/CBP function in neoplastic cells will trigger growth arrest or apoptosis.

### Protein arginine methyltransferases

To date, cancer therapies targeting histone modifications have been directed primarily toward lysine methyltransferases, such as DOT1L and EZH2 ^44^. More recently, the PRMTs have emerged as promising drug targets as well. PRMTs are ubiquitous enzymes and include PRMT1, PRMT4, PRMT5, and PRMT6. Type I PRMTs result in asymmetric arginine dimethylation, whereas type II enzymes catalyze symmetric arginine dimethylation. Most pre-clinical work at this time is focused on PRMT1 (type I), PRMT4 (type I), and PRMT5 (type II). PRMT4, also known as coactivator-associated arginine methyltransferase 1 (CARM1), is a transcriptional regulator with unique histone (H3R17 and H3R26) and non-histone (MLL, splicing factors, SWI/SNF, p300, and RUNX1) targets ^45^. PRMT4 has been shown to be overexpressed in AML cell lines and patient samples and to block myeloid differentiation, possibly through its effects on RUNX1 ^46^. In pre-clinical models, PRMT4 short hairpin RNA (shRNA) knockdown has been shown to induce apoptosis and differentiation of AML cells. Further studies, with small-molecule PRMT4 inhibitors, are ongoing in AML cell lines and murine models. PRMT5 inhibitors are also under development for potential use in lymphoma, solid tumors, and AML ^45, 47, 48^. The PRMTs, particularly PRMT4 (but also PRMT1 and PRMT5), represent an exciting new epigenetic drug target class with the potential to help overcome the myeloid differentiation block and inhibit the aberrant self-renewal seen in AML cells.

## Emerging clinical approaches for acute myeloid leukemia in 2017

Four new drugs were FDA approved for AML in 2017: CPX-351, enasidenib, midostaurin, and GO ( Table 1). These therapies range from targeted single agents (enasidenib, which targets mutant IDH2 [mIDH2] AML) and multi-kinase inhibitors (midostaurin) to a fixed drug combination for induction chemotherapy (CPX-351) and the re-approval of an antibody–drug conjugate that targets CD33 (GO). First, we will briefly review the indications and data behind these new approvals and then we will focus on promising investigational agents and combinations still in development. To induce more durable remissions in patients with AML, particularly for those with relapsed or high-risk disease, we clearly need to initiate rational combination strategies earlier in drug development.

**Table 1.** Recently approved and investigational agents in acute myeloid leukemia and related myeloid neoplasms.

Target	Drug(s)	Approved indication ^a^	Safety
N/A	CPX-351	New dx AML-MRC or t-AML	Myelosuppression and hemorrhage
CD33	GO	Multiple	Myelosuppression and VOD
Mutant FLT3	Midostaurin, sorafenib, gilteritinib, and others	New dx FLT3 ^+^ AML in combination with standard induction ^b^	GI and rash
Mutant IDH2	Enasidenib	R/R IDH2 ^+^ AML	GI and indirect hyperbilirubinemia
Mutant IDH1	Ivosidenib, FT-2102, and others	No	GI
BCL2	Venetoclax	No ^c^	Myelosuppression and GI
LSD1	TCP, IMG-7289, and others	Yes ^d^/No	Thrombocytopenia and neuropsychiatric
Nedd8	Pevonedistat	No	Transaminitis
Mutant TET2	Vitamin C and HMAs	No/Yes ^e^	?
BET	Multiple	No	GI and metabolic

^a^US Food and Drug Administration (FDA) approved indication for acute myeloid leukemia (AML). ^b^Only midostaurin is FDA approved for AML. Sorafenib is FDA approved for other malignancies. Use in AML is off-label but is supported by National Comprehensive Cancer Network (NCCN) guidelines (hypomethylating agent [HMA] combination). ^c^Venetoclax is FDA approved for chronic lymphocytic leukemia (CLL). AML use is off-label at this time. ^d^TCP (tranylcypromine) is FDA approved, but use in AML is off-label. ^e^Azacitidine approved for only myelodysplastic syndrome (MDS), chronic myelomonocytic leukemia (CMML), and low-blast AML in the US (although NCCN guidelines support use in AML patients who are not candidates for induction chemotherapy). In Europe, there are similar approvals for azacitidine and decitabine, but they include all AML. TET2-mutant patients may have increased response rates to HMAs ^19^. TP53-mutant AML patients may have increased response rates to decitabine 10-day schedule ^49, 50^. AML-MRC, acute myeloid leukemia with myelodysplasia-related changes; BCL2, B-cell leukemia/lymphoma-2; BET, bromodomain and extra terminal; Dx, diagnosis; IDH, isocitrate dehydrogenase; GI, gastrointestinal; GO, gemtuzumab ozogamicin; LSD1, lysine-specific demethylase 1; R/R, relapsed/refractory; VOD, (hepatic) veno-occlusive disease.

## Newly approved therapies for acute myeloid leukemia

### CPX-351

CPX-351 is a dual drug liposomal encapsulation containing a 1:5 molar ratio of daunorubicin/cytarabine, which appears to optimize synergy between the two drugs and promote leukemia cell killing in pre-clinical models ^51^. Phase I–III trials in AML have demonstrated that the pharmacokinetics of CPX-351 are dramatically different from conventional bolus dose anthracycline and infusional cytarabine (3+7) and that prolonged drug exposure is demonstrable in patient plasma for 24 hours ^52, 53^. For induction, CPX-351 is given over 90 minutes for three doses on days 1, 3, and 5 (each dose contains 44 mg/m ^2^ of daunorubicin and 100 mg/m ^2^ of cytarabine) and this approach confers more prolonged and continuous exposure to daunorubicin than traditional 3+7. CPX-351 liposomes also appear to accumulate preferentially in the marrow relative to other tissues ^54– 56^.

In a randomized phase III study of 309 patients, Lancet *et al*. showed that CPX-351 conferred a superior overall survival (OS) advantage compared with traditional 3+7 in older patients (60–75 years old) with newly diagnosed secondary AML (defined as t-AML or evolved from a prior hematologic disorder or the presence of MDS-related cytogenetic abnormalities) ^57^. Median OS was superior for CPX-351 versus 3+7 (9.6 versus 5.9 months; *p* = 0.005). CPX-351-treated patients had higher complete remission (CR) rates (38% versus 26%), and more patients in the CPX-351 arm were transplanted in CR1. Patients treated with CPX-351 had increased BM toxicity (delayed count recovery) and increased bleeding events compared with those receiving 3+7, including more fatal central nervous system hemorrhage (2% versus 0.7%). Whether the length of aplasia was the cause of the bleeding risk is not yet fully understood. In contrast, there was less colitis and diarrhea and, anecdotally, less hair loss in the CPX-351 arm, possibly reflecting less extramedullary exposure and toxicity-related CPX-351. It is important to realize that the FDA-approved label for CPX-351 includes all adults (≥18 years old) with t-AML or AML with myelodysplasia-related changes (AML-MRC) and is not limited to older patients. AML-MRC includes patients with a prior diagnosis of MDS or CMML, MDS-related cytogenetic changes, and (while not included in the phase III trial) significant morphologic dysplasia (≥50%) in at least two cell lineages. The optimal clinical role of CPX-351 in the treatment of patients with AML remains to be determined, but, given the CR and OS data, CPX-351 may become the new standard of care for older patients with t-AML/AML-MRC.

### Enasidenib (IDH2 inhibitor)

Enasidenib (AG-221) is a selective, oral inhibitor of the mIDH2 enzyme. Approximately 12% of patients with AML have these mutations ^58^. In the setting of mIDH2, the oncometabolite D-2-hydroxyglutarate (2-HG) is generated rather than αKG. 2-HG inhibits TET protein activity, resulting in downstream DNA hypermethylation and a block in myeloid differentiation similar to the MDS phenotype of TET2 loss of function. The neomorphic mIDH2 enzyme is a promising drug target, and after much pre-clinical work ^59, 60^, the first-in-human phase I/II study of an IDH2 inhibitor (enasidenib), conducted by Stein *et al*. (including our center), showed an overall response rate (ORR) of 40.3% and a CR rate of about 20% (24.4% for IDH2-R172 and 17.7% for IDH2-R140 isoforms) in relapsed/refractory AML patients (176 patients, many heavily pre-treated) ^61^. The results of this dose escalation/expansion study led to the recent FDA approval of enasidenib for relapsed/refractory AML with mIDH2. The primary mechanism of response has been confirmed to be through myeloid differentiation ^62^, and a clinically significant but manageable differentiation syndrome can occur in these patients (7%) ^61^, similar to what has been more frequently observed in APL. Otherwise, the drug is generally well tolerated and grade 1–2 nausea/anorexia and indirect hyperbilirubinemia (grade 3–4 in 12%) are the most frequent side effects. The drug is approved at 100 mg orally daily. The dose was allowed to be increased to 150 or 200 mg daily for lack of response in the phase II portion of the study, but this would currently be off-label usage (of note, the maximum tolerated dose was not reached in the phase I portion at doses beyond 200 mg daily). The average duration of response is about 6 months, but there are some excellent responders with continuous remissions for more than 2 years. Interestingly, in most patients achieving CR, the IDH2 mutant clone persists in functioning neutrophils ^62^. While mIDH2 variant allele frequency (VAF) and the extent of 2-HG suppression do not appear to impact response rates, total mutational burden and the co-occurrence of RAS mutations have been associated with a *decreased* response to IDH2 inhibition ^62^. Combinations of enasidenib with induction chemotherapy in newly diagnosed mIDH2 AML patients ^63^ and with HMAs in older/unfit newly diagnosed or relapsed/refractory mIDH2 AML patients are ongoing ^64^ (ClinicalTrials.gov Identifier: NCT02719574).

### Midostaurin (FLT3 inhibitor)

Midostaurin is a multi-targeted kinase inhibitor active in AML patients with an *FLT3* mutation. The drug was recently approved by the FDA on the basis of the randomized, placebo-controlled phase III RATIFY trial (Randomized AML Trial In FLT3 in patients less than 60 Years old) conducted in younger (18–59 years old) newly diagnosed AML patients with an *FLT3*-ITD or *FLT3*-tyrosine kinase domain ( *FLT3*-TKD) mutation ^65, 66^. Patients (n = 717) were randomly assigned (1:1) to receive midostaurin 50 mg orally twice daily or placebo on days 8–21 of standard induction (3+7 with 60 mg/m ^2^ daunorubicin) and consolidation (high-dose cytarabine). This was a large study and cooperative effort that screened over 3,000 patients, and patients were stratified according to *FLT3* mutation subtype (ITD versus TKD) and ITD-to-wild-type allele ratio (high versus low). Patients were allowed to receive allogeneic HSCT, and those who did not receive an allotransplant received maintenance midostaurin. There was improved OS and event-free survival (EFS) in the midostaurin-treated patients. The difference in median OS (74.7 versus 25.6 months; one-sided *p* = 0.009) appears quite large in favor of the midostaurin group; however, owing to the inflection points on the Kaplan-Meier curves, the authors acknowledged that the hazard ratio (HR) for death likely more accurately reflects the true clinical benefit (HR = 0.78, one-sided *p* = 0.009) (that is, a 22% reduction in death for the midostaurin-treated patients). This survival benefit persisted in patients censored for allogeneic HSCT, reminding us that some FLT3 patients, especially in the midostaurin era, can be cured without transplant. The adverse events occurring in both groups were expected and consistent with those patients receiving intensive chemotherapy experience. The only significant differences in grade 3 or higher toxicity between groups were anemia and rash, which were more common with midostaurin, and nausea, which was less common with midostaurin. Hematologic recovery of neutrophils and platelets were the same between groups. Lastly, it is important to note that patients did well in this study overall; 359 (50.1%) of 717 patients with *FLT3* mutations survived at a median follow-up of 59 months. Whether this was due to better supportive care, improvements in transplantation, or patient selection bias on clinical trials remains to be seen.

### Gemtuzumab ozogamicin

GO is an antibody–drug conjugate that targets CD33, a cell surface antigen that is expressed on the blast cells in 90% or more of patients with AML ^67^. The antibody is covalently linked to calicheamicin, a potent DNA-damaging agent. Most of the cytotoxic payload remains conjugated to the antibody until it is internalized and cleaved by hydrolytic enzymes, but circulating free drug does pose a risk of extramedullary toxicity. GO was initially licensed by the FDA in 2000 for relapsed AML at a dose of 9 mg/m ^2^ for two doses on days 1 and 14 (single agent) but was withdrawn from the market in 2010 because of the risk of hepatic veno-occlusive disease (VOD), particularly in patients who previously had or went on to receive allogeneic HSCT ^68^. The risk of VOD was related to the time elapsed between GO and transplantation or vice versa ^69^. Since then, however, the drug has been further studied, primarily in Europe, at reduced, fractionated doses. With these modifications, several groups have now demonstrated satisfactory safety and efficacy data, leading to the drug’s re-approval in the US in 2017. Adverse side effects include myelosuppression, hepatotoxicity, tumor lysis syndrome (TLS), and infusion reactions. The drug now has three approved indications for AML on the basis of the following three studies:

1. ALFA-0701 study: this 271-patient randomized phase III study examined the addition of GO to standard induction and consolidation chemotherapy (3+7) in patients with newly diagnosed *de novo* AML ^70^. The primary endpoint of median EFS was significantly longer in the GO versus the no GO arm: 17.3 versus 9.5 months ( *p* ≤0.001). VOD occurred in 3% of patients in the GO arm and in none receiving standard of care.

2. AML-19 study: this was a randomized phase III study examining single-agent GO induction (and maintenance) in 237 previously untreated AML patients not candidates for intensive chemotherapy (median age of 77 years) ^71^. Patients treated with GO had improved OS, and the HR for death was 0.69 ( *p* = 0.005). The CR + complete response with incomplete blood recovery (CRi) rate with GO was 27%, and the clinical benefit rate—including partial remission (PR) and stable disease of more than 30 days—was 56.7%. Patients treated with GO experienced myelosuppression, but there was no excess non-hematologic toxicity—including febrile neutropenia, VOD, infection, and bleeding—making this a viable treatment option for older patients.

3. MyloFrance-1 study: this non-randomized 57-patient phase II study evaluated single-agent GO re-induction in *de novo* AML patients with intermediate-risk (78%) or poor-risk (22%) cytogenetics in first relapse (prior HSCT excluded) ^72^. Patients received one course of GO 3.5 mg/m ^2^ on days 1, 4, and 7 (fractionated and lower cumulative dosing compared with prior studies in this population, which used 9 mg/m ^2^ on days 1 and 14). The CR rate in this study was 26%, and median relapse-free survival in these (CR2) patients was 11.6 months. Hematologic toxicity was expected and tolerable, and there was no grade 3 or greater liver toxicity and no VOD, including in patients who went on to receive HSCT.

The FDA has re-approved GO for three indications on the basis of the three studies above, which as a whole used fractionated and lower doses of GO than did the trials that led to its initial approval in 2001. However, GO may add to myelotoxicity during induction, and there is still a risk of VOD, particularly peri-transplant, so caution should be taken with its use. The optimal dose and schedule of GO are still being assessed, particularly in combination with induction chemotherapy, and less may be better, particularly in patients with core-binding factor (CBF) abnormalities ^73, 74^.

## Novel investigational strategies

Some of the most promising novel therapies in AML include small-molecule inhibitors of epigenetic enzymes, inhibitors of anti-apoptotic proteins such as the B-cell leukemia/lymphoma-2 (BCL2) family, and immunotherapy, such as PD-1 axis inhibitors, bispecific antibodies to CD3 and CD123 (expressed in the majority of AML cells), and CAR-T cell approaches.

### Venetoclax

Venetoclax (ABT-199) is a highly selective oral inhibitor of the anti-apoptotic protein BCL2 and is approved for treatment-resistant 17p-deleted chronic lymphocytic leukemia. AML stem and progenitor cells depend on BCL2 for survival, and pre-clinical studies have demonstrated significant activity in AML models ^75^. Clinically, venetoclax was first examined as a single agent in a phase II study of 32 patients with AML (relapsed/refractory or unfit for conventional chemotherapy) ^76^. The venetoclax dose was increased in a stepwise fashion to mitigate the risk of TLS (20 mg on day 1, 50 mg on day 2, 100 mg on day 3, 200 mg on day 4, 400 mg on day 5, and 800 mg on day 6 and thereafter), and TLS did not occur in this study. The ORR in this study was 19% (two CRs and four patients with CRi), all occurring in previously treated patients. Another six (19%) patients had a significant (≥50%) reduction in BM blast percentage. More recently, venetoclax has been combined with HMA therapy ^77^ and low-dose ara-C (LDAC) ^78^ in treatment-naïve older patients (not candidates for induction) with high response rates and apparent safety, although data are still emerging. A recent phase Ib study of 57 patients treated with venetoclax HMA combination showed a 61% combined CR/CRi rate ^77^. Given the impressive response rates of about 60 to 70% observed with both HMA and LDAC combinations, venetoclax has received breakthrough therapy designation by the FDA to accelerate its development as a frontline combination partner for older/unfit AML patients (both HMA and LDAC combinations).

### Other FLT3 inhibitors

The FLT3 receptor tyrosine kinase drives cellular proliferation and survival through the JAK-STAT, MEK/MAPK, and PI3K signaling pathways. Both ITD and point mutations in the TKD occur in AML, resulting in constitutive activity of the enzyme. *FLT3*-ITD is one of the most common mutations in AML (~30%) and is associated in general with a poor prognosis ^79– 81^. Typically, patients have a high white blood cell count at presentation and are chemo-sensitive, usually achieving CR. However, relapse usually occurs without allogeneic HSCT, and patients rapidly become chemo-refractory. Co-occurring *NPM1* mutations somewhat ameliorate the prognosis of *FLT3*-ITD AML ^82^, placing patients in a more intermediate-risk category, whereas co-occurring *DNMT3A* mutations or trisomy 8 worsen the prognosis, as does a high *FLT3*-ITD allele burden, particularly in the absence of mutated *NPM1* ^9, 82^. *FLT3*-TKD mutations (usually D835) occur in an additional 10% of patients (approximately), and the prognostic impact is less clear and is likely not as unfavorable as the ITD. Given the rapidly fatal nature of relapsed FLT3-mutant AML, FLT3 inhibitors have been in development for almost two decades. While FLT3 inhibitors have been shown to have clinical activity in AML and can serve as a bridge to allo-HSCT, there are still no approved FLT3 inhibitors for *relapsed*/ *refractory* FLT3-AML. Sorafenib is a multi-kinase inhibitor that inhibits FLT3 along with vascular endothelial growth factor receptor and other kinases, and it is approved for hepatic, thyroid, and kidney cancer. Sorafenib is often used off-label in combination with azacitidine for relapsed/refractory or older/unfit FLT3-AML, given the lack of other good options for these patients outside of a clinical trial, and this use is supported by National Comprehensive Cancer Network guidelines based on a phase II trial of 37 evaluable patients with FLT3-AML showing an ORR of 46% with 10 CRi, six CR, and one PR ^83^. Median duration of response in this study was 2.3 months (range of 1–14.3 months), underscoring the fact that the emergence of treatment resistance to FLT3 inhibition is a frequent problem. Sorafenib has also shown promise as a maintenance therapy for FLT3-ITD-mutant AML post-transplant, and retrospective data show that it improves OS (HR 0.26, *p* = 0.021) ^84^. Other studies of newer-generation FLT3 inhibitors such as quizartinib, crenolanib, and gilteritinib are ongoing or completed. A problem with most FLT3 inhibitors is that, while they may be transiently effective against the FLT3-ITD and may serve as a bridge to allo-HSCT in some patients, acquired resistance-conferring point mutations at D835 (or other activation loop substitutions) or gatekeeper (F691) mutations commonly occur over a period of months, resulting in loss of response. Gilteritinib, an oral FLT3 and AXL inhibitor, was recently reported to have activity against some of these mutations. A phase I/II study in relapsed/refractory FLT3-mutated AML showed a 57.5% overall response rate and 47.2% composite CR rate—CR, CRi, and CR with incomplete platelet recovery (CRp)—in 106 patients receiving doses of 80 mg daily or higher ^85^. A phase III registration trial with gilteritinib is ongoing in relapsed/refractory FLT3-AML.

### IDH1 inhibitors

Like the *IDH2* gene, the *IDH1* gene is mutated in AML. IDH1 resides primarily in the cytosol, whereas IDH2 is primarily mitochondrial. The phenotype of *IDH1* or *IDH2* mutant AML is similar, characterized by the gain-of-function, neomorphic mutant IDH activity producing 2-HG, which in turn impairs cellular differentiation. *IDH1* mutations (IDH1m) are slightly less common than IDH2 mutations in AML; the former are seen in about 8 to 10% of patients, mostly those with normal karyotype AML, and the most commonly co-occurring mutations are in the *NPM1*, *DNMT3A*, and *FLT3* genes ^86^. *IDH1* and *IDH2* mutations are mutually exclusive to each other and to TET2 and WT1 mutations. The first study using an IDH1 inhibitor (AG-120), a large phase I/II study, has now completed enrollment; response rates to AG-120 appear to be similar to those to enasidenib (AG-221). In the first 78 evaluable MDS/AML patients (most relapsed/refractory), the overall response rate was 38.5% with 14 CRs (17.9% of patients) ^87^. The side effect profile of AG-120 is similar to that of enasidenib (that is, it too is well tolerated). As with the IDH2 inhibitor, most patients achieving CR with AG-120 did not achieve IDH1 mutation clearance by next-generation sequencing (VAF ≥1%, median coverage 500×). As with IDH2 inhibitor therapy, differentiation-like syndrome can occur with IDH1 inhibitors, and it may be more common in patients receiving concomitant HMA therapy. Multiple studies of different IDH1 inhibitors are ongoing, including newer-generation IDH1 inhibitors and combinations of these agents (or AG-120) with HMAs and induction chemotherapy.

### LSD1 inhibitors

In addition to IDH inhibitors, other new drugs targeting epigenetic modifications have strong therapeutic potential. Epigenetic enzymes such as DNMTs, histone deacetylases (HDACs), and histone methyltransferases/demethylases all represent bona fide targets for anti-cancer drug development and several have been discussed herein ^40, 88^. It is important to remember that epigenetic events play an essential role in promoting myeloid leukemogenesis and that epigenetic alterations occur far more frequently than genetic mutations in AML blasts. Treatment with DNA demethylating agents leads to clinical responses in about 20 to 30% of cases ^89^. Two HMAs (azacitidine and decitabine) are well known and approved for MDS, CMML, and low-blast AML in the US and Europe (and are also approved for older adults with more than 30% blasts in Europe). However, histone methylation and DNA methylation both play critical roles in transcriptional regulation. Notably, specific histone methyl marks coordinately regulate chromatin structure to activate (for example, H3K4me2) or inactivate (for example, H3K27me2) myeloid differentiation programs. Specifically, the removal of H3K4me2 is catalyzed by lysine-specific demethylase 1 (LSD1), which removes mono- and di-methyl marks on H3K4. LSD1 is highly expressed in patients with AML. Removal of these marks by LSD1 generally interferes with myeloid differentiation; therefore, inhibiting LSD1 may result in the restoration of myeloid gene expression, although this may be cell context dependent. LSD1 is being actively investigated as a drug target; LSD1 inhibitors have shown anti-leukemic activity (generally cytostatic) in pre-clinical models and may function as differentiating agents ^90, 91^. Early phase single-agent LSD1 inhibitor clinical trials are ongoing, and further studies combining LSD1 inhibitors with HMAs are being designed.

In addition, LSD1 inhibitors have promising therapeutic potential when given in combination with ATRA. ATRA has revolutionized the treatment of APL, a subtype of AML (about 5–10% of all AML) characterized by the PML-RARα (PML-RARA) fusion protein, arising from a balanced translocation involving the *RARA* gene on chromosome 17 and the *PML* gene on chromosome 15. When given in combination with chemotherapy or arsenic trioxide, ATRA induces differentiation and ultimately cure in virtually all APL patients who do not die from early bleeding events ^92^. However, ATRA has little, if any, effect on differentiation or anti-leukemic activity in patients with non-APL AML. One of the underlying reasons for this lack of response to ATRA in non-APL AML is the failure to induce transcriptional activation of target genes such as *RARA*. However, LSD1 inhibition in combination with ATRA appears to unlock these transcriptional programs ^93^. Therefore, several LSD1 inhibitors are currently being studied with ATRA, including tranylcypromine (TCP) (Parnate ^®^), an irreversible monoamine oxidase (MAO) inhibitor that is FDA approved as an antidepressant and anxiolytic agent. TCP has more recently been shown to inhibit LSD1, decreasing H3K4 methylation ^94^, and Schenk *et al*. have demonstrated that, when added to ATRA, TCP greatly potentiates the differentiation response in non-APL AML cell lines and primary patient samples, leading to decreased leukemia burden and elimination of leukemia-initiating cells ^93^. Given the availability of TCP, it has already been re-purposed in combination with ATRA for phase I clinical trials in both the US and Europe, which are currently accruing. More potent LSD1 inhibitors than TCP are being studied pre-clinically and have demonstrated greater differentiative effect in combination with ATRA ^95^.

### BET bromodomain inhibitors

Bromodomain (BRD) and extra terminal (BET) family proteins are epigenetic readers that bind acetylated histones and facilitate the transcription of various oncogenes, such as *c*- *Myc*, primarily by regulating their super-enhancers. BRD4 inhibitors are being studied in different clinical trials in a variety of solid-organ and hematologic malignancies, including AML (ClinicalTrials.gov Identifier: NCT02711137). MYC is overexpressed in most AML samples (~70%), as are other BRD-regulated pathways such as Ras and Hedgehog ^96^. Pre-clinically, BRD inhibition has activity in AML cell lines, including FLT-ITD-mutated and MLL-rearranged cells, as well as in mouse xenograft models ^97^. Several clinical trials are ongoing in myeloid neoplasms, including combinations with HMAs ^98^.

### Targeting mutant TP53

The TP53/chromosomal aneuploidy subtype of AML represents about 10 to 20% of AML ^8^ and is enriched in older patients, many of whom have antecedent MDS or t-AML ^10^. TP53 alterations are common but not always present in complex karyotype AML (60%) and predict for even worse prognosis within this subgroup ^99^. Although CPX-351 has been approved for secondary-type AML (p53 status not known on the phase III trial) as previously discussed and may affect outcomes in this subgroup going forward, patients with TP53/aneuploidy historically fare extremely poorly even with allogeneic HSCT ^99^. Decitabine has shown some promise to bridge these patients to allo-HSCT, particularly the 10-day regimen, with high rates of blast clearance, but long-term outcomes with decitabine remain to be seen, and it has not been compared head-to-head with standard induction chemotherapy ^49, 100^.

New agents are being developed for this very poor-risk subtype of AML. The neddylation (Nedd8) inhibitor pevonedistat has shown recent promise in this subgroup ^50^. Neddylation is a process similar to ubiquitination, is catalyzed by Nedd8-activating enzyme (NAE), and is required for cullin-ring ligase (CRL)-mediated degradation of various protein substrates. Inhibiting the polymerization of Nedd8 breaks down this process, resulting in the accumulation of cytotoxic substrates within the cell and apoptosis. In a phase I dose-escalation study of pevonedistat plus azacitidine in treatment-naïve older AML patients, the combination was well tolerated, ORR was 50% (20 CR, five CRi, and seven PR), and 63% of responders (20/32) responded during the first two cycles ^101^. In addition, four out of five patients with *TP53* mutations responded (CR/PR) with two maintaining response for more than 10 cycles. A randomized phase II study of pevonedistat (at the RP2D of 20 mg/m ^2^ intravenously on days 1, 3, and 5 of a 28-day cycle) with or without azacitidine (standard dose and schedule) in the same patient population has completed enrollment (ClinicalTrials.gov Identifier: NCT02610777), and a phase III study is ongoing (ClinicalTrials.gov Identifier: NCT03268954). In addition, for myeloid neoplasms with *TP53* point mutations, refolding agents (for example, APR-246) to restore the wild-type conformation of the TP53 protein are being studied in clinical trials (ClinicalTrials.gov Identifier: NCT03072043).

## Conclusions

Improved understanding of the genetic and epigenetic alterations underlying AML development have refined prognostic algorithms and are starting to generate better treatment options for patients. Recently approved drugs have demonstrated improved outcomes in AML, and this is likely just the beginning as we continue to develop more individualized approaches to treat the different subtypes of AML with combinations of chemotherapy, kinase inhibitors, and epigenetic-targeted therapies. Recent therapeutic advances have largely resulted from improved delivery of chemotherapy with CPX-351 and GO. Mutation-targeted therapies for AML have also emerged with IDH2 and FLT3 inhibitors. The future of AML therapy lies in the rational combination of these agents, informed by pre-clinical research and selected for the right patient, and early phase clinical trials should incorporate more combinatorial and biomarker-driven arms early in their development.
